# Supplementary material for: How to make climate-neutral aviation fly
Source: Nat Commun. 2023 Jul 6;14:3989. doi: 10.1038/s41467-023-39749-y (PMC10326079; doi:10.1038/s41467-023-39749-y)
Supplement: Supplementary file 1 — Supplementary Information [file 41467_2023_39749_MOESM1_ESM.pdf]

Supplementary information document for:

How to make climate-neutral aviation fly

Romain Sacchi*$1, Viola Becattini*2, Paolo Gabrielli2, Brian Cox3, Alois Dirnaichner4, Christian Bauer1, Marco Mazzotti$2

** These authors contributed equally to this work*
$ Corresponding authors: email romain.sacchi@psi.ch*; email* marco.mazzotti@ipe.mavt.ethz.ch

1 Technology Assessment group, Laboratory for Energy Systems Analysis, Paul Scherrer Institut, Villigen, Switzerland
2 Institute of Energy and Process Engineering, ETH Zurich, Zurich, Switzerland
3 INFRAS, Bern, Switzerland
4 Potsdam Institute for Climate Impact Research, Potsdam, Germany

---

Results for the 3.5° C climate scenario

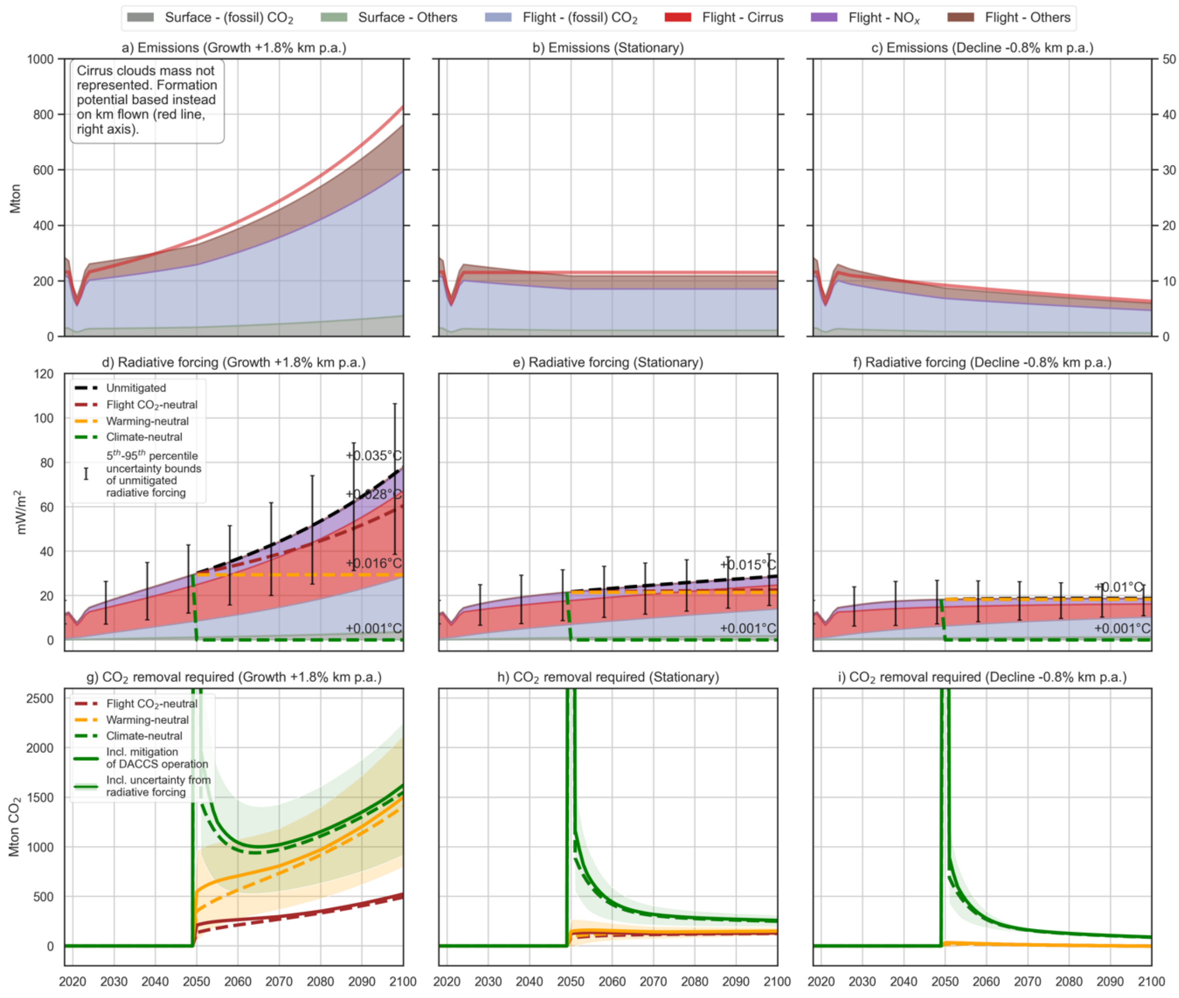

Fig. S.1: European aviation fleet activity for 2018-2100 relying on jet fuel and Carbon Dioxide Removal (CDR) performed via Direct Air Capture and Carbon Storage (DACCS) to meet the mitigation scope, for the three *air-traffic demand trajectories,* under the 3.5° C climate scenario. Panels a), b) and c): amount of climate forcers emitted (left y-axis) and of kilometers flown (right y-axis); Panels d), e) and f): Radiative Forcing (RF) of climate forcers and forcing trajectories for the

mitigation scopes considered (note: the additional RF caused by DACCS is calculated iteratively, and included in the total RF, see Supplementary Information); Panels g), h) and i): CDR requirement (with and without additional removal needed to mitigate DACCS operations) to meet a given mitigation scope over the 2050-2100 period. Error bars represent the uncertainty around the radiative efficiency of flight SLCF emissions.

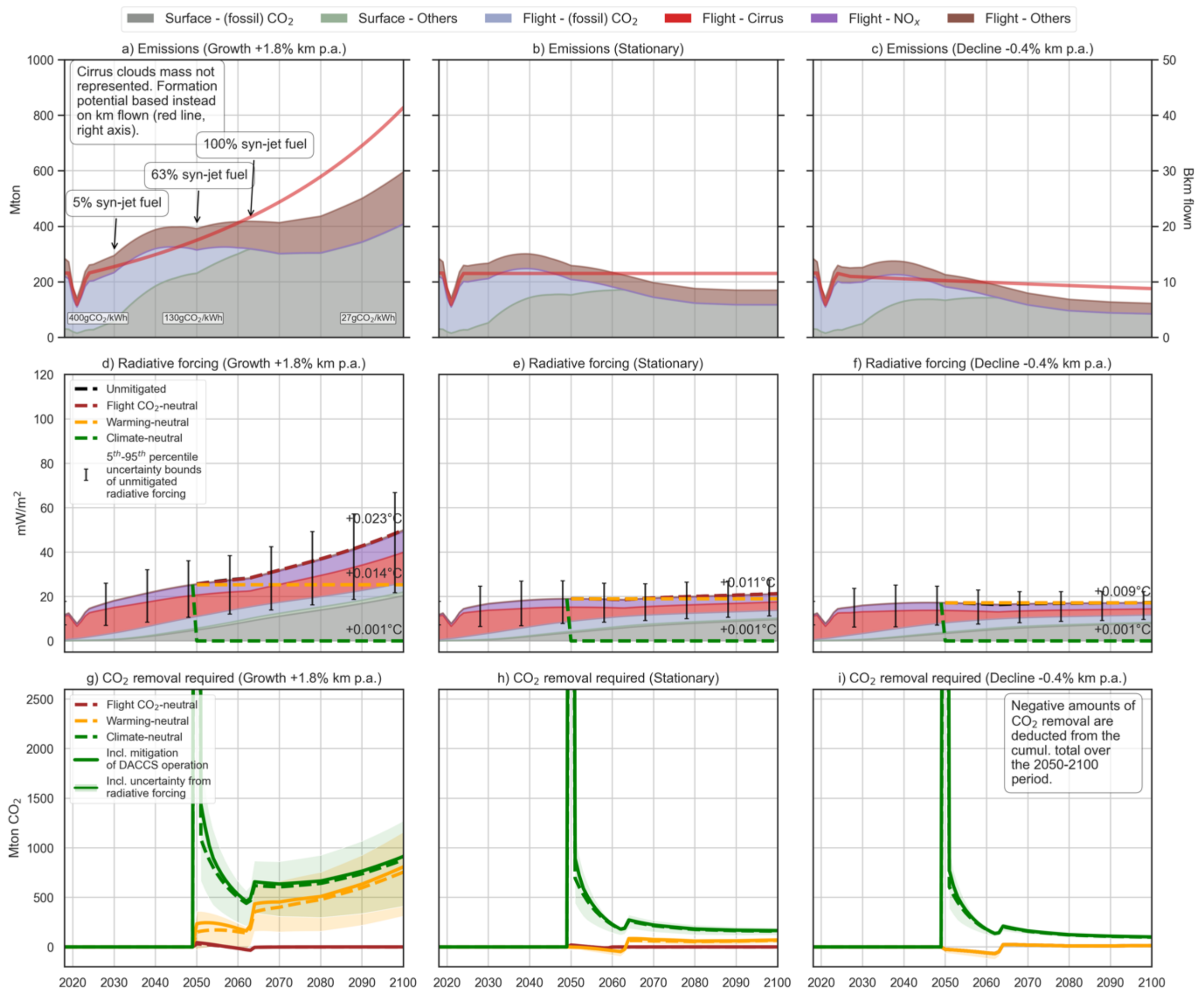

Fig. S.2: European aviation fleet activity for 2018-2100 relying on syn-jet fuel and Carbon Dioxide Removal (CDR) performed via Direct Air Capture and Carbon Storage (DACCS) to meet the mitigation scope, for the three *air-traffic demand trajectories,* under the 3.5° C climate scenario. Panels a), b) and c): amount of climate forcers emitted (left y-axis) and kilometers flown (right y-axis). The CO_2 intensity of electricity along the time axis is indicated at the bottom and is provided by the climate scenario; Panels d), e) and f): Radiative Forcing (RF) of climate forcers and forcing trajectories for the mitigation scopes considered (note: the additional RF caused by DACCS is calculated iteratively and included in the total RF, see Supplementary Information); Panels g), h) and i): CDR requirement (with and without additional removal needed to mitigate DACCS operations) to meet a given mitigation scope over the 2050-2100 period. Error bars represent the uncertainty around the radiative efficiency of flight SLCF emissions.

Air traffic demand trajectories

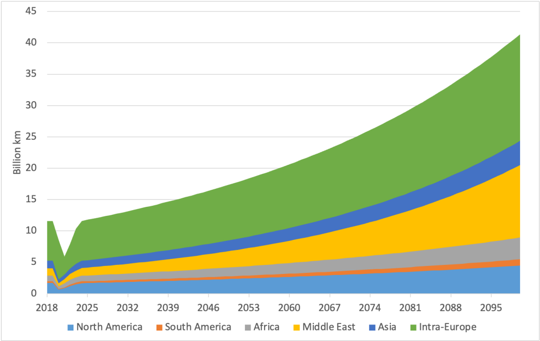

a) Growing demand trajectory (+1.8% p.a.)

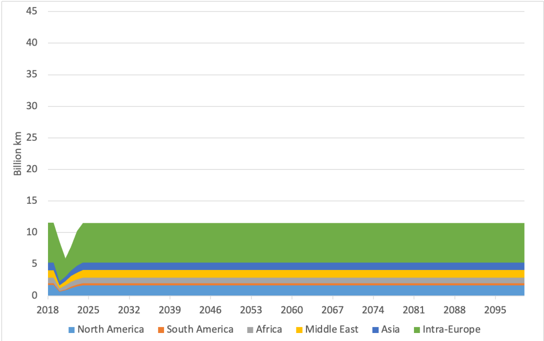

b) Stationary demand trajectory

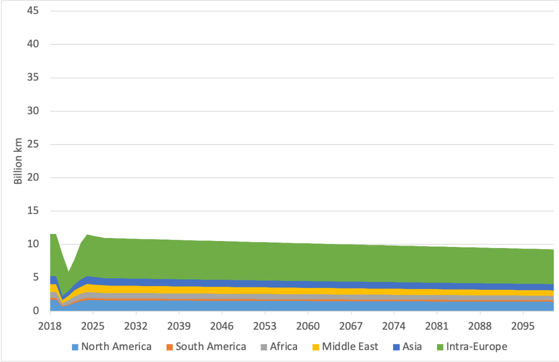

c) Declining demand trajectory (-0.2% p.a.) for a fleet using syn-jet fuel in the 2° C climate scenario.

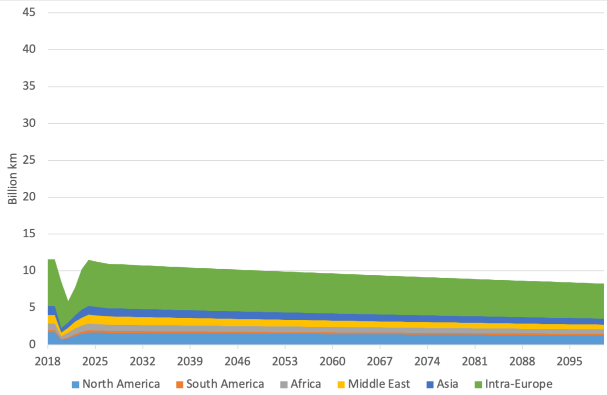

d) Declining demand trajectory (-0.4% p.a.) for a fleet using syn-jet fuel in the 3.5° C climate scenario.

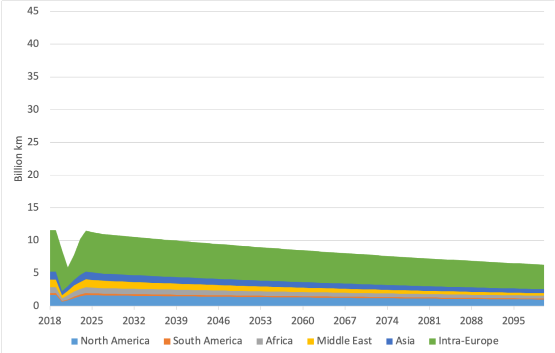

e) Declining demand trajectory (-0.8% p.a.) for a fleet using (fossil) jet fuel.

Fig. S.3: In billions of kilometers flown, demand trajectories for the European passenger fleet by destination.

Sensitivity analyses

This section presents an analysis to show the sensitivity of results presented in Figure 4 on several model parameters for the scenario aiming at climate neutrality, with a growing European air-traffic demand trajectory, in a 2 °C climate scenario (<u>blue diamonds</u> in Figure 4, referred to in the following as "*reference scenario*").

The following cases are considered for the sensitivity analysis:

- (A) *10 €/MWh by 2050*. In this scenario, the price of electricity used throughout the life-cycle phases of the aircraft fleet (i.e., including the production of syn-jet fuel and the operation of the DAC system followed by storage of CO_2 underground) converges at a value of 10 € per MWh in 2050 and remains at this level until 2100. By contrast, the price of electricity assumed in the *reference scenario* is 56€/MWh, as indicated by the global model REMIND v.3.2 using the SSP2 RCP 2.6 scenario for the European region.
- (B) *100 €/MWh by 2050*. Similarly to case (A) above, the price of electricity converges at a value of 100 € per MWh in 2050 and remains at this level until 2100. The price of electricity assumed in the *reference scenario* is 56€/MWh.
- (C) *Fuel burn -2 %/year*. The fuel burn rate, in liters of kerosene per seated passenger per 100 km, is the product of engine efficiency, seating capacity, and occupancy rate and changes as these parameters vary over time. In this scenario (C), it decreases by 2% annually between 2018 and 2050, which corresponds to the aspirational goal of the ICAO[1]. In the *reference scenario*, the fuel burn rate is assumed to decrease by 1.2% annually between 2018 and 2050 – and remains constant until 2100.
- (D) *+2.5%/year*. In this scenario, the fleet grows at a rate of 2.5% annually (i.e., in terms of kilometers flown) between 2025 and 2100. In the *reference scenario*, the annual fleet growth rate is assumed to be 1.8% in the same period.
- (E) *Reduced RF for cirrus clouds*. Based on a recent estimate by Digby et al.[2], the radiative efficiency index associated with forming cirrus clouds is decreased to 14% of the value used in the *reference scenario* (i.e., 1.31^{-10} mW/m^2/km flown instead of 9.36^{-10} mW/m^2/km flown, as suggested earlier by Lee et al. [3]).
- (F) *100% nuclear*. The electricity used to power the different systems throughout the life-cycle of the aircraft fleet (i.e., including the production of syn-jet fuel and the operation of the DAC system followed by storage of CO_2 underground) originates from nuclear power, with a carbon intensity of 6 grams of CO_2 per kWh, to which various transmission and distribution-related losses apply. In the *reference scenario*, the carbon intensity of electricity in 2018 is 400 grams of CO_2 per kWh, gradually reduced to 30 grams in 2100. We do not change the price of electricity.

- (G) *No atm. benefits from syn-jet fuel*. In this scenario, the exhaust emission factors related to the combustion of syn-jet fuel are considered similar to those of fossil-based jet fuel, including emissions of sulfur oxides and volatile organic compounds. Additionally, we do not consider any reduction in the formation of ice particles and the related decrease in cloudiness and persistence of cirrus clouds associated with the use of syn-jet fuel, as described in Voigt et al.[4]. In the *reference scenario*, we assume that the use of syn-jet fuel reduces the number of ice particles by 88%, leading to a 65% reduction in the radiative forcing of cirrus clouds formation.
- (H) *Lower targets for syn-jet fuel*. The penetration rate of syn-jet fuel in Europe is revised downwards. In the *reference scenario*, we adopt the targets of the EU ReFuel initiative[5], which concerns sustainable aviation fuels in general (i.e., including biomass-based jet fuel), assuming that the content of syn-jet fuel in the blend would increase to 63% in 2050. In this scenario (H), we consider the sub-targets specific to syn-jet fuel instead. These targets are 0.7% of the fuel blend in 2030, 8% in 2040, 11% in 2045, and 28% in 2050. We extrapolate this trajectory (+2.8% annually) to reach 100% in 2076 (as opposed to 2063 in the *reference scenario*).

The results of the sensitivity analyses are illustrated in Figure S1, where the amounts of the five resources needed between 2018 and 2100 for the *reference scenario* (blue diamonds, Figure 4) and for the cases (A) to (H) are shown on the left-hand side of each figure for the use of jet fuel and on the right-hand side for the use of syn-jet fuel. In the *reference scenario*, larger amounts of all resources – except for geological CO_2 storage – are needed when using syn-jet fuel; much larger amounts of electricity, land, and freshwater, with slightly higher costs. These trends are generally confirmed in all the six scenarios above, where the same comparison can be made, i.e., cases (A) to (F), as (G) and (F) are specific to syn-jet fuel; this is true when the resource demand considered both increases and decreases in the new scenario compared to the *reference scenario*. There are only a few exceptions. Regarding Costs, scenarios (A) and (C) have the potential to make both fuel options equally expensive, either because of the fall of the electricity price to 10 €/MWh by 2050 or the decrease of the fuel burn rate by 2% annually between 2018 and 2100. Also, regarding geological CO_2 storage, under scenario (G), the syn-jet fuel option requires a storage capacity close to that of the fossil jet fuel option since there are no benefits in terms of reduced radiative forcing from the limited cloudiness and persistence of cirrus clouds when using syn-jet fuel. These sensitivity analyses show that the conclusions drawn from Figure 4 in Main about the demand for resources remain valid because they are robust with respect to the variation of some of the key model parameters.

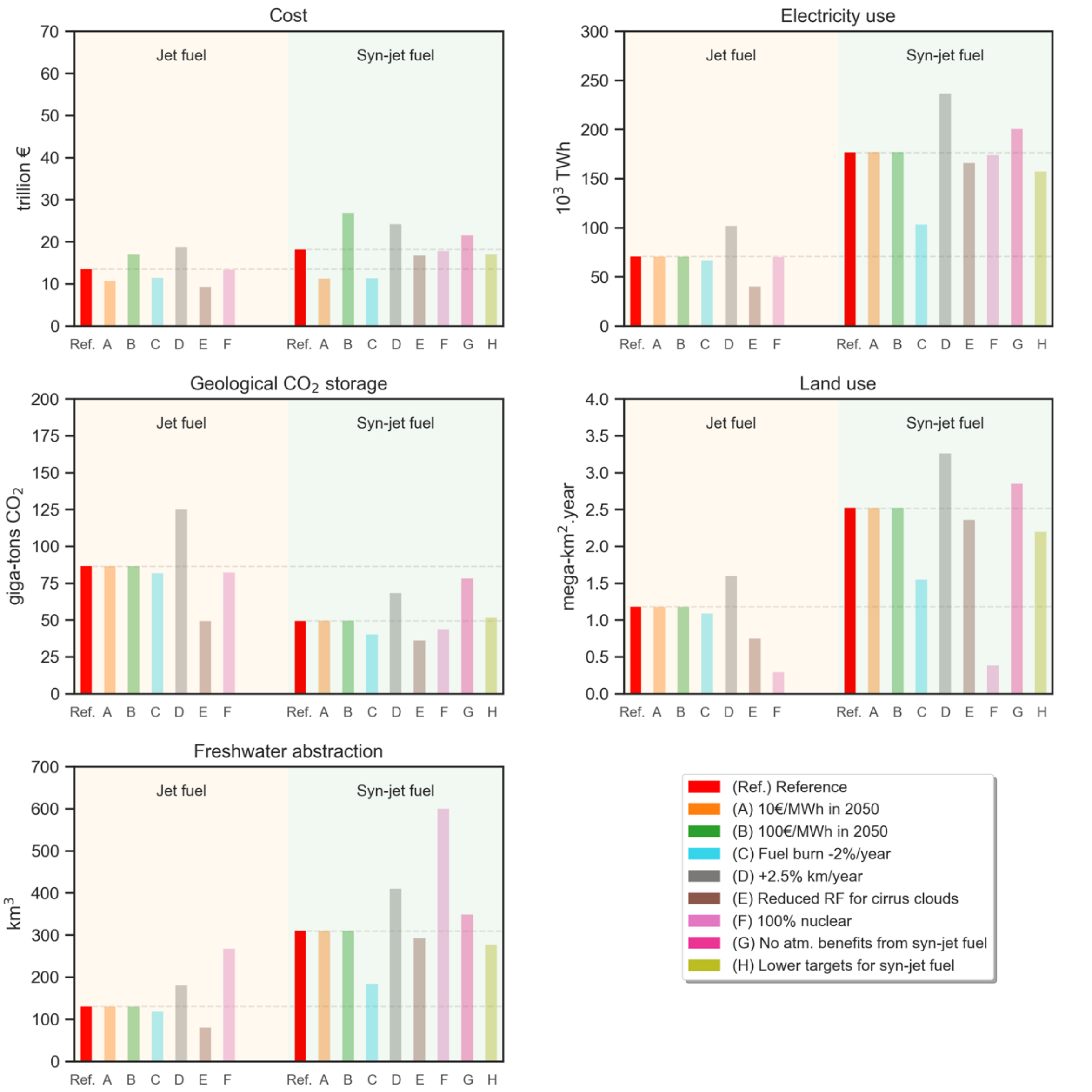

Figure S.4 Sensitivity results. Ref. = *reference scenario*, i.e., results presented in Figure 4 (blue diamonds) of Main with reference to a growth trajectory in air-traffic demand, a climate-neutral mitigation strategy, in a +2 °C climate scenario.

References

1. Fleming, G. G., Ivan de Lépinay & Roger Schaufele. *Environmental Trends in Aviation to 2050 Environmental Trends in Aviation to 2050 Background*. https://www.icao.int/sustainability/Pages/Post-Covid-Forecasts-Scenarios.aspx (2019).

2. Digby, R. A. R., Gillett, N. P., Monahan, A. H. & Cole, J. N. S. An Observational Constraint on Aviation-Induced Cirrus From the COVID-19-Induced Flight Disruption. *Geophys. Res. Lett.* **48**, e2021GL095882 (2021).

3. Lee, D. S. *et al.* The contribution of global aviation to anthropogenic climate forcing for 2000 to 2018. *Atmos. Environ.* **244**, 117834 (2021).

4. Voigt, C. *et al.* Cleaner burning aviation fuels can reduce contrail cloudiness. *Commun. Earth Environ. 2021 21* **2**, 1–10 (2021).

5. European Commission. Sustainable aviation fuels – ReFuelEU Aviation. https://ec.europa.eu/info/law/better-regulation/have-your-say/initiatives/12303-Sustainable-aviation-fuels-ReFuelEU-Aviation_en (2020).
